# Development and Application of a Mechanistic Cooling and Freezing Model of the Spin Freezing Step within the Framework of Continuous Freeze-Drying

**DOI:** 10.3390/pharmaceutics13122076

**Published:** 2021-12-03

**Authors:** Gust Nuytten, Susan Ríos Revatta, Pieter-Jan Van Bockstal, Ashish Kumar, Joris Lammens, Laurens Leys, Brecht Vanbillemont, Jos Corver, Chris Vervaet, Thomas De Beer

**Affiliations:** 1 Laboratory of Pharmaceutical Process Analytical Technology, Department of Pharmaceutical Analysis, Faculty of Pharmaceutical Sciences, Ghent University, 9000 Ghent, Belgium; PieterJan.VanBockstal@UGent.be (P.-J.V.B.); Laurens.leys@ugent.be (L.L.); 2 Escuela Profesional de Química, Facultad de Ciencias, Universidad Nacional de Ingeniería, Puerta 5—Av. Tupac Amaru N° 210 Rimac, Lima 15333, Peru; susanrios25@gmail.com; 3 Pharmaceutical Engineering Research Unit, Department of Pharmaceutical Analysis, Faculty of Pharmaceutical Sciences, Ghent University, 9000 Ghent, Belgium; ashish.kumar@ugent.be; 4 Laboratory of Pharmaceutical Technology, Department of Pharmaceutics, Faculty of Pharmaceutical Sciences, Ghent University, 9000 Ghent, Belgium; joris.lammens@ugent.be (J.L.); Chris.Vervaet@UGent.be (C.V.); 5 Coriolis Pharma, Fraunhoferstraße 18 b, 82152 Martinsried, Germany; brechtvanbillemont@gmail.com; 6 RheaVita, Frieda Saeysstraat 1, 9052 Zwijnaarde, Belgium; j.corver@rheavita.com

**Keywords:** continuous freeze-drying, spin freezing, mechanistic model, uncertainty analysis, global sensitivity analysis

## Abstract

During the spin freezing step of a recently developed continuous spin freeze-drying technology, glass vials are rapidly spun along their longitudinal axis. The aqueous drug formulation subsequently spreads over the inner vial wall, while a cold gas flow is used for cooling and freezing the product. In this work, a mechanistic model was developed describing the energy transfer during each phase of spin freezing in order to predict the vial and product temperature change over time. The uncertainty in the model input parameters was included via uncertainty analysis, while global sensitivity analysis was used to assign the uncertainty in the model output to the different sources of uncertainty in the model input. The model was verified, and the prediction interval corresponded to the vial temperature profiles obtained from experimental data, within the limits of the uncertainty interval. The uncertainty in the model prediction was mainly explained (>96% of uncertainty) by the uncertainty in the heat transfer coefficient, the gas temperature measurement, and the equilibrium temperature. The developed model was also applied in order to set and control a desired vial temperature profile during spin freezing. Applying this model in-line to a continuous freeze-drying process may alleviate some of the disadvantages related to batch freeze-drying, where control over the freezing step is generally poor.

## 1. Introduction

**Figure 1:**
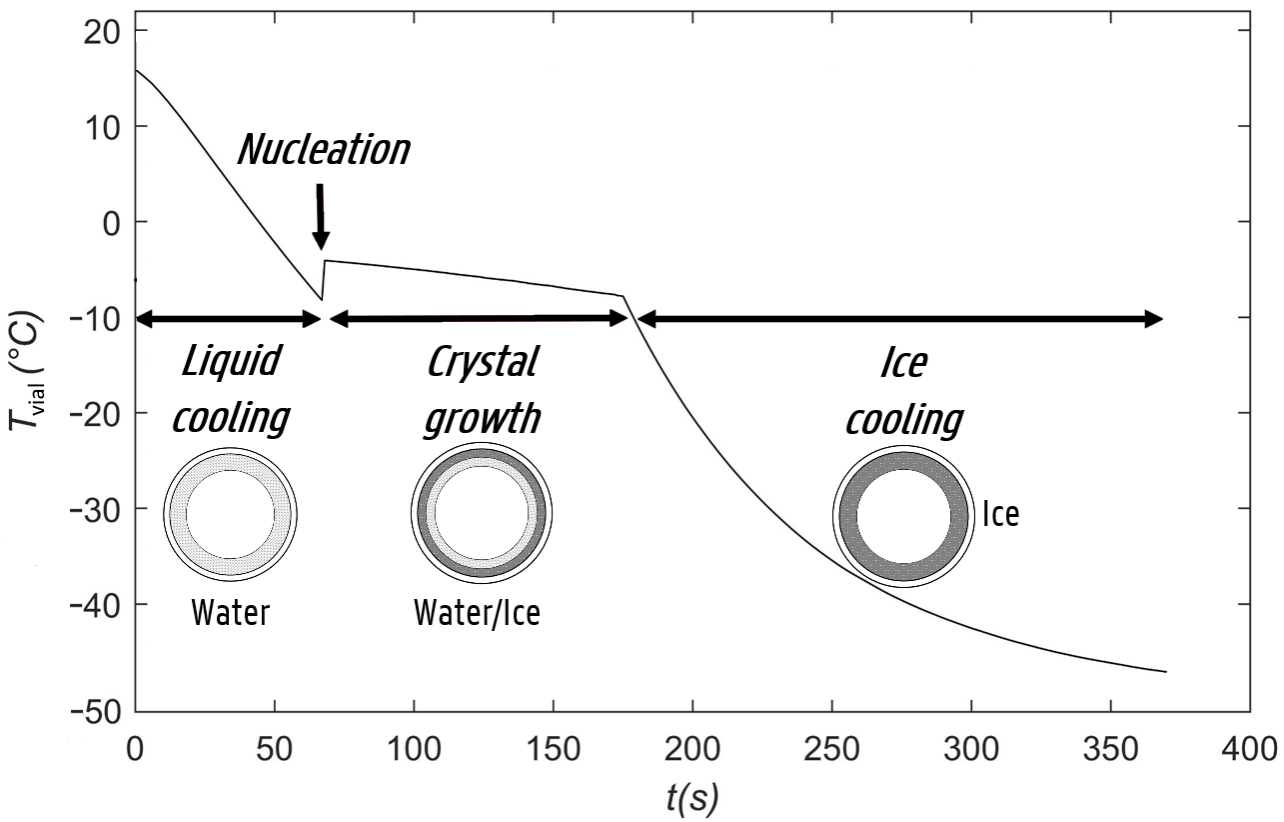
Diagram of vial temperature over time during the separate phases of cooling and freezing. The process starts with the cooling of the vial containing liquid product, until the nucleation temperature is reached. At this point, the temperature of the product within the vial increases up to the equilibrium temperature, and the ice crystallization phase starts. This phase ends when all water has crystallized into ice, whereafter the vial containing the now-solid product cools down until the final temperature. A cross-section of the vial during spin freezing is shown.

Freeze-drying or lyophilization is a low-temperature drying method under vacuum conditions used for aqueous drug solutions with poor stability [1]. Freeze-drying is divided in three sequential steps: freezing, primary drying, and secondary drying. Freezing is the initial step during which the aqueous solution is solidified, allowing sublimation of the ice and desorption of unfrozen water in the subsequent drying steps. First, the solution is cooled down until ice nucleation occurs, i.e., the moment when the first ice crystal is formed, generally at a temperature several degrees below the equilibrium freezing temperature. The exothermic nature of ice crystallization results in an instantaneous increase in product temperature to the equilibrium freezing temperature (see below, Figure 1). Further cooling of the product leads to further exothermic ice crystal growth, characterized by a slowly decreasing overall product temperature as a result of a growing ice layer. The product temperature decreases sharply once the aqueous solution is completely frozen (see below, Figure 1). The freezing step is considered complete when the product temperature reaches a sufficiently low value that allows for further product processing with a minimal risk of exceeding the eutectic temperature ($T_{e}$) or the glass transition temperature of the maximum freeze-concentrated solution ($T\prime {}_{g}$).

The ice nucleation temperature and the kinetics of ice crystal growth determine the physical state and morphology of the frozen solution and, consequently, some of the final properties (e.g., the pore size distribution) of the freeze-dried product [2]. Ice nucleation is a stochastic phenomenon that is related to process and product properties such as container surface roughness and the amount of suspended particles present in the solution [3,4]. Due to the stochastic nature of ice nucleation, freezing in a conventional batch freeze-drying process presents significant disadvantages, as it causes uncontrolled product quality variations from vial to vial within the same batch and between batches. Therefore, much effort has been made to develop methods for controlled nucleation in batch freeze-drying, with the aim of achieving a more similar degree of supercooling for all vials [2,5,6,7,8,9,10,11,12,13]. The degree of supercooling is defined as the difference between the equilibrium freezing temperature and the temperature at which the first ice nucleus is formed (i.e., primary nucleation) [2,13]. Secondary nucleation follows primary nucleation, and secondary ice nuclei are formed until the equilibrium temperature is reached [4]. A higher degree of supercooling results in a larger amount of heat freed by exothermic crystallization upon ice nucleation. Hence, a larger fraction of the water is instantaneously frozen, resulting in the formation of more ice nuclei during secondary nucleation compared to ice nucleation at a higher temperature (i.e., a lower degree of supercooling). It is expected that the amount of ice nuclei determine the amount of resulting ice crystals after complete solidification, as during solidification the existing ice nuclei grow, but no new ice nuclei are formed [14]. In this way, a lower degree of supercooling generally leads to a smaller amount of larger ice crystals, resulting in larger pores in the dried product layer during freeze-drying [13]. Additionally, the temperature-controlled shelves most often cool down at a set constant rate until a final temperature during freezing. In this way, more time will have passed before nucleation in the case of a high degree of supercooling compared to a low degree of supercooling. Hence, in the case of high supercooling the shelves will be colder at the moment of nucleation compared to low supercooling. The product equilibrium temperature will remain unchanged (e.g., at 0 °C in the case of pure water) in both cases, resulting in a larger temperature difference between shelf and product in the case of higher supercooling. This increased temperature gradient is associated with faster heat removal during ice-crystal growth, yielding ice crystals with a different morphology (i.e., needle-like ice crystals for fast heat removal and dendritic crystals for slow heat removal) [14]. This difference in ice-crystal morphology may lead to relevant differences in the dried product characteristics (e.g., dried product resistance to water vapour). In essence, for batch freeze-drying, the uncontrolled freezing and the stochastic nature of ice nucleation inherently causes vial-to-vial variability in product characteristics within a batch and between batches [13].

Taking the above into account, it is evident that a good understanding and control of the freezing step and its different phases (i.e., liquid cooling, nucleation, ice crystallization, and subsequent solid cooling) during freeze-drying is of importance. In practice, model-based approaches are often utilized to gain process knowledge and to develop control mechanisms [3,15,16,17,18,19]. Several approaches have been proposed to model the freezing step in batch freeze-drying, such as the one developed by Nakagawa et al. [3,15]. They proposed a two-dimensional axisymmetric cooling and freezing model based on the conductive heat equation (i.e., Fourier’s law of heat conduction). The modelling was divided into two phases: the cooling step of the aqueous solution before ice nucleation, followed by the freezing step including the nucleation event and ice-crystal growth.

The cooling step was simulated using the conductive heat equation expressed in Equation (1) [3,15]. This equation describes the conductive heat flux in relation to the physical properties of the product (i.e., the mass density, the specific heat capacity, and the thermal conductivity), as well as the temperature distribution of the product under study.

(1) $$\rho c_{p}\left(\frac{\delta T}{\delta t}\right)=\nabla \left(k\nabla T\right)$$

Here, ρ is the solution mass density (kg/m^3^); $c_{p}$ describes the specific heat capacity of the solution (J/(kg K)); *k* is the thermal conductivity (W/(m K)); and ∇T is the temperature distribution of the system (K).

Afterwards, the freezing step starts at the moment ice nucleation occurs until ice-crystal growth is completed. The second modelling phase is based on the same base equation but includes two source terms corresponding to the total latent heat released due to ice nucleation, $Q_{n}$, and the total latent heat released due to ice crystallisation, $Q_{c}$, as given in Equation (2) [3,15].

(2) $$\rho c_{p}\left(\frac{\delta T}{\delta t}\right)=\nabla \left(k\nabla T\right)+Q_{n}+Q_{c}$$

The positive source term $Q_{n}$ was estimated as follows:

(3) $$Q_{n}=\Delta H_{f}k_{i}\left(T_{eq}-T_{s}\right)$$

where $\Delta H_{f}$ represents the heat released due to ice crystallisation (J/kg); $k_{i}$ describes the nucleation rate (kg/(m^3^ s K)); $T_{eq}$ is the equilibrium freezing temperature (K); and $T_{s}$ is the temperature in the supercooled liquid (K).

The nucleation rate $k_{i}$ (kg/(m${}^{3}$ s K)) was calculated using the freezing front velocity ν (m/s), the thickness of the undercooled zone *s* (m), and the homogeneous undercooled temperature T′ (K). [3]:

(4) $$k_{i}=\frac{\rho \nu }{s(T_{eq}-T^{\prime })}$$

The positive-source term $Q_{c}$ corresponding to the latent heat of crystallization was estimated using the following equation:

(5) $$Q_{c}=\Delta H_{f}\frac{\delta (\rho \chi_{ice})}{\delta t}$$

where $\chi_{ice}$ represents the ice fraction, which is a suspension of ice in the liquid water phase [3].

The mean ice crystal size L* (m) was estimated using the following equation:

(6) $$L*=aR^{-0.5}G^{-0.5}$$

with *a* as an empirical constant based on experimental data (ms/K), *R* as the freezing front rate (m/s), and *G* as the temperature gradient in the frozen zone (K/m).

Based on the above equations, freezing curves for aqueous solutions were calculated and compared to experimental temperature data, and a good agreement was found. The authors determined the ice-crystal growth rates and the temperature gradients in the aqueous solution, to estimate the ice crystal mean sizes and the resulting water vapour mass transfer permeability. Their results showed that water vapour mass transfer resistance decreases as the nucleation temperature increases and the cooling rate decreases. This was in accordance with the ice crystal size estimation obtained from image analysis. The author concluded that the freezing conditions have a direct impact on the permeability of the dried layer during the sublimation step [3]. This model described the data well and provided important insights into the freezing process. However, in order to apply their model to next-generation technologies such as spin freezing [1], several additions need to be made.

In this work, a mechanistic cooling and freezing model using elements of the model by Nakagawa et al. and applied to the spin freezing step of a continuous freeze-drying technology was developed. During continuous freeze-drying, a constant inflow of vials filled with an aqueous formulation are rapidly rotated along their longitudinal axis, while a flow of a cold, inert, and sterile gas is used for the cooling and freezing of the product. These spin-frozen vials are further processed in the consecutive continuous drying steps, making use of radiative heat [1,20]. This continuous freeze-drying method has several advantages compared to batch freeze-drying. Importantly, similar processing conditions are created for all vials, and the process can be monitored using in-line PAT tools and controlled at a single vial level [21]. The freezing step in particular can be controlled well, since the flow rate and temperature of the cooling gas can be set in order to control the rate of heat transfer during the entire freezing process, as will be shown in this work. The goal of this work was the development and application of a mechanistic model to predict and control the vial temperature during the spin freezing step using thermal imaging. The developed model was evaluated thoroughly by global sensitivity and uncertainty analyses, as well as experimentally.

## 2. Materials and Methods

### 2.1. Spin Freezing

**Figure 2:**
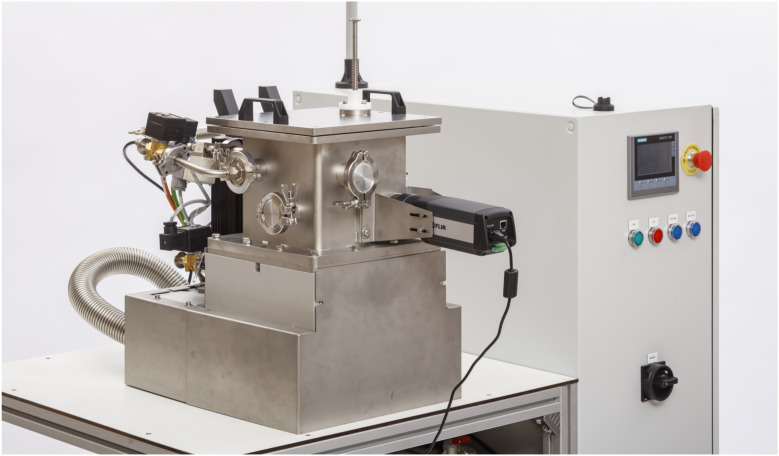
Single-vial continuous freeze-drying system.

**Figure 3:**
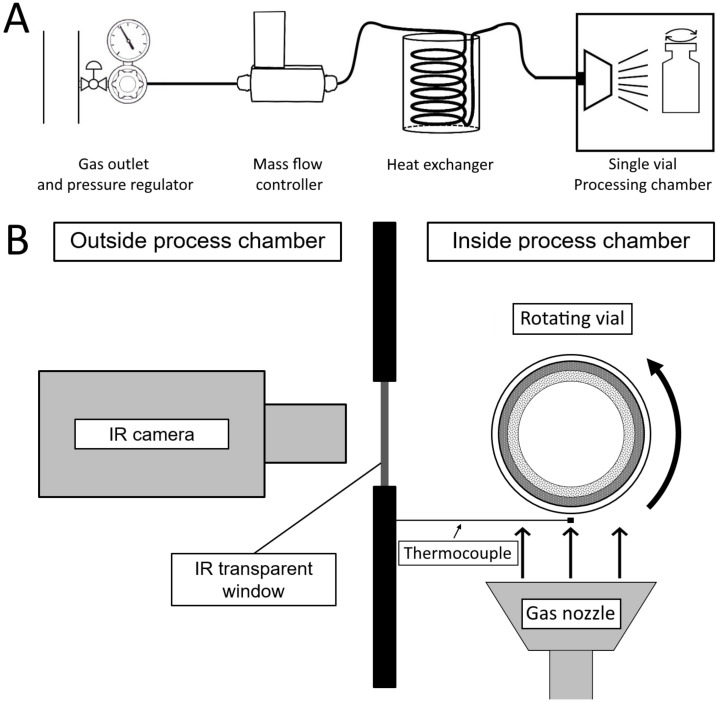
Schematic representation of the cooling system during spin freezing. (**A**): Scheme showing how pressurized air travels from the gas outlet through a pressure regulator, after which the gas flow rate is subsequently determined by a mass flow controller. The pressurized air then passes through the liquid nitrogen heat exchanger before flowing through the gas diffuser and cooling the rotating vial within the single-vial processing chamber. (**B**): Top view of the experimental setup, showing the rotating vial, the outlet of cold gas from the gas nozzle, the gas temperature measurement thermocouple, and the IR camera located outside the chamber measuring through a germanium window.

All spin freezing experiments were conducted in a single-vial continuous freeze-dryer (RheaVita, Zwijnaarde, Belgium) (Figure 2). Ten mL type I glass vials (Schott, Müllheim, Germany) were filled with 3.0 mL of demineralized water. The vial was placed in a holder supported with grippers inside the stainless-steel processing chamber. The vial was rotated around its longitudinal axis at approximately 2900 rpm. Simultaneously, dehumidified air, cooled using a heat exchanger, was jetted onto the vial. The heat exchanger consisted of polyurethane tubing with an outer diameter of 8 mm and an inner diameter of 5 mm. Three m of this tubing was inserted into a Dewar container that was filled with liquid nitrogen. The cooled air passed through a stainless steel gas diffuser and travelled 1.5 cm before hitting the rotating vial. The cooling gas flow rate was measured and controlled using a Bronkhorst® F-203AV digital mass flow controller (Flowcor, Belgium). The temperature of the cooling gas was measured using a type K thermocouple positioned between the rotating vial and the outlet of the gas diffuser, at a distance of 1 mm from the rotating vial. The temperature of the vial was continuously followed up using a FLIR A655sc IR camera (Thermal Focus, Ravels, Belgium) as described in previous work [21]. The IR camera was installed outside the chamber at a distance of 20 cm from the vial, in front of an IR-transparent germanium window (Figure 3). The transmission of the germanium window was 0.86, and the emissivity of the measured borosilicate vial glass was 0.93. The vial temperature, the gas temperature, and the gas flow-rate data were simultaneously logged every 0.50 s and collected using a custom-made Labview® virtual instrument. The recording of data started as soon as the gas flow rate stabilized, usually within 4 s of starting the gas flow.

It should be noted that when the gas temperature changes, the thermocouple does not instantly report the correct new value, as the thermocouple has to equilibrate with the new gas temperature. This is of particular importance in this work, as the gas temperature has a substantial impact on the cooling process and changes rapidly at the start of the process. Indeed, at the start of spin freezing, the cooling gas is still relatively warm due to heating from the cooling system (e.g., the tubing the gas flows through, which is initially at room temperature). However, the system rapidly cools along with the gas temperature, necessitating a measurement lag correction. A lag error $T_{e,lag}$ (K) was defined and calculated by the following equation [22]:

(7) $$T_{e,lag}=-N_{t}\frac{dT_{gas}}{dt}$$

with $\frac{dT_{gas}}{dt}$ as the rate of change of the gas temperature (K/s) and $N_{t}$ as the time constant (s).

The time constant $N_{t}$ is defined as the ratio of the thermal sensor heat capacity to the thermal conductance of the thermal sensor material. Majdak et al. developed an empirical least-squares regression model that allows calculation of $N_{t}$ based on the type of thermocouple used, which was applied in this research to the used thermocouple type and dimensions. The following equation was used [23]:

(8) $$N_{t}=\frac{1}{N_{t,a}+N_{t,b}\sqrt{w}}$$

Here, $N_{t,a}$ (1/s) and $N_{t,b}$ (m${}^{-\frac{1}{2}}$s${}^{-\frac{1}{2}}$) are regression model parameters that depend on the type of thermocouple used, and *w* is the air flow velocity (m/s).

Using $T_{e,lag}$, $T_{gas}$ was corrected at every timepoint by subtracting the lag error.

### 2.2. Mechanistic Cooling and Freezing Model

The mechanistic cooling and freezing model developed during this study predicts the temperature profile of the outer-vial wall for every discrete time step of the spin freezing process. From the outer-vial wall temperature, the product temperature and temperature at the inner vial wall can be calculated as described below. In our developed model, the spin freezing step was divided into four phases: liquid cooling, ice nucleation, crystal growth, and solid cooling (Figure 1). Liquid cooling refers to the phase where the vial contents are present in the liquid state and cooling of this system (i.e., the vial including its contents) takes place. The liquid cooling stage ends at the moment ice nucleation occurs and the first ice crystals are formed. The subsequent crystal growth phase refers to the transition from the remaining liquid to the solid state. Finally, solid cooling refers to further cooling of the completely solidified product, down to the final freezing temperature where the model simulation ends.

In the developed spin freezing model, the rate of heat transfer $\dot{Q}$ (W) is used to describe the amount of energy per time unit that is removed from the system. $\dot{Q}$ is calculated at every time point of the spin freezing process using Newton’s law of cooling [24]:

(9) $$\dot{Q}=\pi h{Ø}_{vial}h_{vial}\left(T_{v,o}-T_{gas}\right)$$

where *h* is the heat transfer coefficient (W/(m${}^{2}$K)); ${Ø}_{vial}$ is the diameter of the vial (m); $h_{vial}$ is the height of the vial (m); $T_{v,o}$ is the temperature at the outside of the vial wall (K); and $T_{gas}$ (K) is the temperature of the cooling gas.

The heat transfer coefficient *h* depends on several factors, including the rotation speed of the vial, the distance between the rotating vial and the gas nozzle, the vial type and dimensions, the longitudinal position of the vial, the properties of the freezing chamber, the gas diffuser properties, the cooling gas properties, the heat-exchanger properties, environmental factors (e.g., ambient temperature around the freezing chamber), and the gas flow rate [25]. However, most of these parameters remain constant during spin freezing. Only the gas flow rate is changed during spin freezing, as this parameter is used to control *h*, and therefore $\dot{Q}$. Hence, a linear regression model (i.e., with the gas flow rate as the independent variable and the heat transfer coefficient as the dependent variable) that is applicable for the certain set of spin freezing conditions mentioned above was created. In this way, *h* can be calculated from the gas flow rate using the regression model, at any point in time.

In order to create the linear regression model, eight calibration experiments were performed at constant flow rates (i.e., 20, 27, 34, 41, 48, 55, 62, and 69 L/min), whereafter the heat transfer coefficient was calculated at every time point for every experiment by rearranging Equation (9) as follows:

(10) $$h=\frac{\bar{Q}}{\pi {Ø}_{vial}h_{vial}\left(T_{v,o}-T_{gas}\right)}$$

Here, the vial dimensions ${Ø}_{vial}$ and $h_{vial}$ were known constants, and $T_{v,o}$ and $T_{gas}$ were measured at every time point. The mean rate of heat transfer $\bar{Q}$ was calculated using the length of the crystal growth phase $t_{cryst}$ according to the following equation:

(11) $$\bar{Q}=\frac{Q_{cryst}}{t_{cryst}}$$

Here, $Q_{cryst}$ is the heat released due to the crystallization of the vial contents (J). The slight decrease in temperature of the solid ice layer during this phase was considered to be negligable with regards to energy transfer, and it was assumed that all heat released due to crystallization is removed at a rate $\bar{Q}$. When the crystal growth is complete, the vial temperature profile displays a marked drop, since the exothermic process of ice crystallization stops. This allows determination of $t_{cryst}$ using vial temperature profile data.

$Q_{cryst}$ was calculated as follows:

(12) $$Q_{cryst}=m_{water}\Delta H_{f}$$

where $m_{water}$ is the mass of water contained in the rotating vial (kg), and $\Delta H_{f}$ is the latent heat released during ice crystallization (J/kg).

The linear regression model was then constructed using the least-squares method, where an average *h* was calculated per calibration experiment (i.e., one value for *h* per gas flow rate) from the heat transfer coefficients calculated at every timepoint (Equation (10)):

(13) $$h=h_{a}\dot{V}+h_{b}$$

Here, $\dot{V}$ is the volumetric gas flow rate (m${}^{3}$/s), $h_{a}$ is the regression coefficient (J/(m${}^{5}$K)), and $h_{b}$ (W/(m${}^{2}$K)) is the regression error term. Using this equation, *h*, and subsequently $\dot{Q}$, were calculated for every timepoint of the process. The root mean square error of cross-validation (RMSECV) was calculated using the leave-one-out method [26].

As mentioned previously, the developed model predicts $T_{v,o}$ during every phase of spin freezing for every discrete time step. Firstly, during the liquid cooling phase, $T_{v,o}$ decreases every time step with a certain amount, according to the following equation:

(14) $$T_{v,o}\left(i\right)=T_{v,o}\left(i-1\right)-\frac{\dot{Q}\left(i-1\right)}{C_{tot,w}}dt$$

Here, $T_{v,o}\left(i\right)$ refers to the temperature at the outer wall of the vial at the current time step; $T_{v,o}\left(i-1\right)$ refers to this temperature at the previous time step; and dt refers to the length of the time step (s). In this way, $T_{v,o}$ decreases every time step with a value of $\frac{\dot{Q}\left(i-1\right)}{C_{tot,w}}dt$, where $\dot{Q}\left(i-1\right)$ refers to the rate of heat transfer of the previous step, and $C_{tot,w}$ is the total heat capacity of the system before crystal growth (J/K). $C_{tot,w}$ depends on the specific heat capacity of the used vial glass $c_{pvial}$ (J/(kg K)), the weight of the vial $m_{vial}$ (kg), the specific heat capacity of water $c_{p\;water}$ (J/(kg K)), and the mass of water contained in the vial $m_{water}$ (kg):

(15) $$C_{tot,w}=c_{pvial}m_{vial}+c_{p\;water}m_{water}$$

The calculation of the decreasing product temperature continues until the product temperature reaches the nucleation temperature $T_{nuc}$. As mentioned above, ice nucleation is a stochastic phenomenon, which is difficult to accurately predict, even with controlled nucleation methods. In this model, nucleation is not predicted and, consequently, was an input for the model. However, it is possible to detect nucleation during spin freezing using in-line thermal imaging, as it is associated with a sharp increase in the vial temperature. At high rates of heat transfer (i.e., at high gas flow rates), it is possible that the developed temperature gradient within the liquid layer becomes sufficiently large so that not all contents of the vial are below $T_{eq}$ at the time of nucleation. Therefore, it is assumed that nucleation then only occurs in the zone of the product where the temperature is below $T_{eq}$. This zone is defined by a volume $V_{nucl}$, calculated as follows:

(16) $$V_{nucl}=\pi h\left(r_{v,i}^{2}-r_{sc}^{2}\right)$$

where $r_{v,i}$ is the inner vial radius (m), and $r_{sc}$ is the radius of ice nucleation (m) outside of which ice nucleation takes place, which is calculated as follows:

(17) $$r_{sc}=r_{v,i}e^{-\frac{2\pi k_{ice}h_{vial}\left(T_{eq}-T_{v,i}\left(i\right)\right)}{\dot{Q}\left(i\right)}}$$

where $k_{ice}$ is the thermal conductivity of ice at 0 ${}^{{}^{\circ}}$C (W/(m K)); $T_{eq}$ (K) is the equilibrium freezing temperature; and $T_{v,i}\left(i\right)$ refers to the temperature at the inner vial wall (K). Here, $T_{v,i}\left(i\right)$ must be calculated from $T_{v,o}\left(i\right)$, by using Fourier’s law of heat conduction adapted to a hollow cylinder [27]:

(18) $$T_{v,i}=T_{v,o}+\frac{\dot{Q}\ln\left(\frac{r_{v,o}}{r_{v,i}}\right)}{2\pi k_{glass}h_{vial}}$$

with $r_{v,o}$ as the outer vial radius (m), and $k_{glass}$ as the thermal conductivity of the vial glass (W/(m K)).

**Figure 4:**
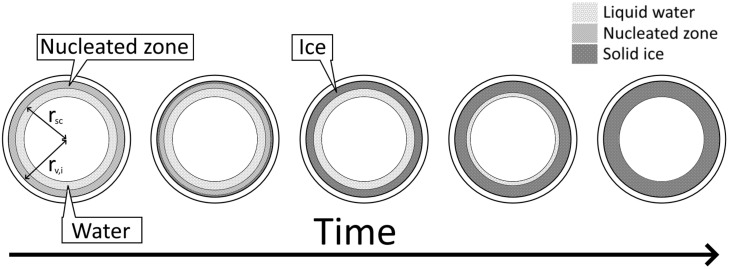
Axial view of spin freezing with ice layer thickness progression from left to right. The ice layer grows over time as more water crystallizes into ice, until all water has solidified and the crystal growth phase is complete. The inner vial radius $r_{v,i}$ and the radius of the nucleated zone $r_{sc}$ are shown.

It was assumed that nucleation occurs homogeneously in the zone of the product between $r_{sc}$ and $r_{v,i}$ [2,28]. An ice nucleation fraction ($\chi_{nuc}$) was calculated at the starting point of crystallization:

(19) $$\chi_{nuc}=\frac{C_{tot,w}\left(T_{eq}-T_{nuc}\right)}{\Delta H_{f}V_{nucl}\rho_{water}}$$

where $\rho_{water}$ (kg/m${}^{3}$) is the water density. From here on, the crystal growth phase of the model starts, where a certain amount of ice crystallizes every discrete time step. Contrary to shelf freezing, during spin freezing, energy is removed from the curved outer surface of the vial, and not from the planar bottom surface. This results in a freezing front that travels centripetally from the edge of the inner glass vial wall towards the centre of the rotating vial (Figure 4). This is in contrast to a circular planar freezing front travelling from the bottom of the vial towards the top, as seen in shelf freezing. The ice crystallizing results in an increase in thickness of the ice layer, as illustrated in Figure 4. Outside of $r_{sc}$, ice grows where ice nuclei are already present, which has to be taken into account:

(20) $$m_{ice}\left(i\right)=m_{ice}\left(i-1\right)+\frac{\dot{Q}\left(i-1\right)\left(1+\chi_{nuc}\right)}{\Delta H_{f}}dt$$

within $r_{sc}$, no ice was present right after nucleation, and the total mass of ice as the freezing front propagates through this layer was calculated as follows:

(21) $$m_{ice}\left(i\right)=m_{ice}\left(i-1\right)+\frac{\dot{Q}\left(i-1\right)}{\Delta H_{f}}dt$$

In both Equations (20) and (21), $m_{ice}\left(i\right)$ refers to the mass of ice present in the current time step, and $m_{ice}\left(i-1\right)$ refers to the mass of ice in the previous time step. Just like in the liquid cooling phase, $\dot{Q}\left(i-1\right)$ refers to the rate of heat transfer in the previous time step. During crystallization, the temperature of the liquid product is assumed to be the equilibrium temperature.

However, to calculate $T_{v,o}$, the temperature gradient across the growing ice layer and the glass wall of the vial must be taken into account. The temperature gradient across the ice layer can again be calculated using Fourier’s law of heat conduction (Equation (18)), with some minor adjustments:

(22) $$\dot{Q}=2\pi k_{ice}h_{vial}\frac{T_{eq}-T_{v,i}}{ln(\frac{r_{v,i}}{r_{v,i}-th_{ice}})}$$

with $k_{ice}$ as the thermal conductivity of ice (W/(m K)). In order to calculate $T_{v,i}$, the ice layer thickness $th_{ice}$ is required, which was calculated according to the following equations:

(23) $$V_{cryst}=\frac{m_{ice}}{\rho_{ice}}$$

(24) $$th_{ice}=r_{v,i}-\sqrt{\frac{\left(\pi r_{v,i}^{2}h_{vial}\right)-V_{cryst}}{\pi h_{vial}}}$$

with $V_{cryst}$ as the volume of the crystallized ice (m${}^{3}$) and $\rho_{ice}$ as the density of ice (kg/m${}^{3}$).

Equation (22) can then be rewritten as:

(25) $$T_{v,i}=T_{eq}-\frac{\dot{Q}\ln\left(\frac{r_{v,i}}{r_{v,i}-th_{ice}}\right)}{2\pi k_{ice}h_{vial}}$$

$T_{v,o}$ should then be calculated from $T_{v,i}$ as before using Equation (18). The ice layer thickness increases until all liquid water has been converted to ice. At this point, the solid cooling phase starts, which is the final phase of the model. This phase is analogous to the liquid cooling phase, but the specific heat capacity of ice $c_{p\;ice}$ (J/(kg K)) instead of water should be taken into account when calculating the total heat capacity:

(26) $$C_{tot,i}=c_{p\;vial}m_{vial}+c_{p\;ice}m_{ice}$$

with $C_{tot,i}$ as the total heat capacity of the frozen system (J/K). In this way, Equation (14) becomes:

(27) $$T_{v,o}\left(i\right)=T_{v,o}\left(i-1\right)-\frac{Q(i-1)}{C_{tot,i}}$$

The spin freezing process ends when the final vial temperature (i.e., the temperature where the model simulation ends) is reached during the solid cooling phase. In this work, the final temperature was set at −50 °C.

### 2.3. Uncertainty Analysis and Global Sensitivity Analysis

An uncertainty analysis (UA) and a global sensitivity analysis (GSA) were conducted in order to characterize the developed mechanistic model. The UA allows quantification of the impact of the uncertainty of the model input parameters on the uncertainty in the model outcome, while the GSA allows to apportion the uncertainty in the model output to different sources of uncertainty in the model parameters [29].

A total of seven parameters were considered to be uncertain and therefore were included in the UA and GSA. These parameters, their uncertainty level, and the reason for their inclusion are listed in Table 1. The uncertainty in the heat transfer coefficient *h* was specified as the root-mean-square error (RMSE) of the linear regression model. The uncertainty of the vial properties was their permissible variation, as obtained from the vial manufacturer. The error on the mass of the water was calculated from the density of water and the accuracy of the pipetted volume, which was obtained from the pipette manufacturer. The uncertainty in the gas flow rate was based on the accuracy of the mass flow controller measurement. The equilibrium temperature was measured using an infrared camera, meaning its uncertainty value was equal to the accuracy of the used infrared camera. Finally, the uncertainty in the gas temperature originates from the accuracy of the used thermocouple.

A sampling-based approach was used for the UA on the freezing model [17,30]. For this method, the model was run for different combinations of input process variables, based on their uncertainty range (i.e., Monte Carlo simulations). The input matrix containing 10,000 samples (i.e., 10,000 different combinations of input parameters) was constructed using the Sobol sampling technique [17,30]. For all of the 10,000 samples, the mechanistic model was applied to predict a vial temperature profile, resulting in 10,000 predicted temperature profiles. In this way, 10,000 temperature values were obtained at every time point (i.e., every 0.5 s) for calculation of the uncertainty limits. At every time point, these 10,000 temperature values were ordered from low to high. The lower temperature limit of the prediction interval was defined as the temperature value corresponding to sample number 250 (i.e., 2.5%), while the upper limit was defined as the value corresponding to sample number 9750 (i.e., 97.5%). This approach was used for every time point of the process, resulting in a lower and an upper uncertainty limit, together forming a 95% prediction interval. Experimental temperature data were subsequently compared to the uncertainty limits.

For the GSA, a variance-based sensitivity analysis was conducted as it involves no prior assumptions about model output and allows quantification of overall interaction effects between factors [16,19]. A total order effect $S_{Ti}$ was calculated from the decomposition of the total output variance into the contribution of the input factors [16,19]. $S_{Ti}$ represents the total effect of factor i, which is the sum of the first-order effects, higher-order effects, and all interactions with other factors. The design proposed by Saltelli et al. was applied to calculate $S_{Ti}$ in this study [29]. The mathematical details regarding the computation of $S_{Ti}$ are described by Mortier et al. [16]. The GSA was conducted at different timings, each matching with a specific phase of the spin freezing process (i.e., the liquid cooling phase, the ice-crystal growth phase, and the solid cooling phase), in order to evaluate the difference in impact of the uncertain model input parameters at each process phase. The GSA was also performed for multiple gas flow rates: at a low gas flow rate (20 L/min), an intermediary gas flow rate (50 L/min), and a high gas flow rate (80 L/min).

### 2.4. Experimental Model Verification of Constant Gas Flow Rate and Imposed Cooling Profile Experiments

The model was verified using the data of 10 spin freezing experiments where the following constant flow rates were used: 20, 26, 32, 38, 44, 50, 56, 62, 68, and 80 L/min. An uncertainty analysis was performed as described above at every flow rate using data (e.g., gas and vial temperature) from the respective experiment.

Additionally, in order to demonstrate its applicability, the model was used to impose a targetted vial temperature profile. This profile was set to have a liquid cooling phase with a cooling rate of 20 ${}^{{}^{\circ}}$C/min, a crystal growth phase with a duration of 150 s, and a solid cooling phase with a cooling rate of 20 ${}^{{}^{\circ}}$C/min. The required rate of heat transfer $\dot{Q}$ to achieve this during liquid and solid cooling was calculated from the cooling rate:

(28) $$\dot{Q}=C_{r}C_{tot}$$

where $C_{r}$ is the cooling rate (K/s), and $C_{tot}$ is $C_{tot,w}$ for the liquid cooling phase and $C_{tot,i}$ for the solid cooling phase. The required rate of heat transfer $\dot{Q}$ during the crystallization phase was calculated using Equations (11) and (12). From $\dot{Q}$, *h* was calculated for every timepoint using Equation (9). Finally, the required cooling gas flow rate was calculated from Equation (13) at every time point, which was then an input for the mass flow controller. The same imposed cooling profile was applied to perform 20 freezing cycles in order to assess the robustness of this model application. An uncertainty analysis was performed on the model vial temperature predictions for one representative replicate of the 20 imposed cooling temperature profile runs (i.e., with a cooling rate of 20 ${}^{{}^{\circ}}$C/min during the liquid cooling phase, a crystal growth phase with a duration of 150 s, and a solid cooling phase with a cooling rate of 20 ${}^{{}^{\circ}}$C/min) and compared to the recorded vial temperature data.

All calculations were performed using the numeric computing platform MATLAB version R2018b (The MathWorks, Natick, MA, USA).

## 3. Results and Discussion

### 3.1. Experimentally Obtained Vial Temperature Profiles and Subsequent Uncertainty Analysis

**Figure 5:**
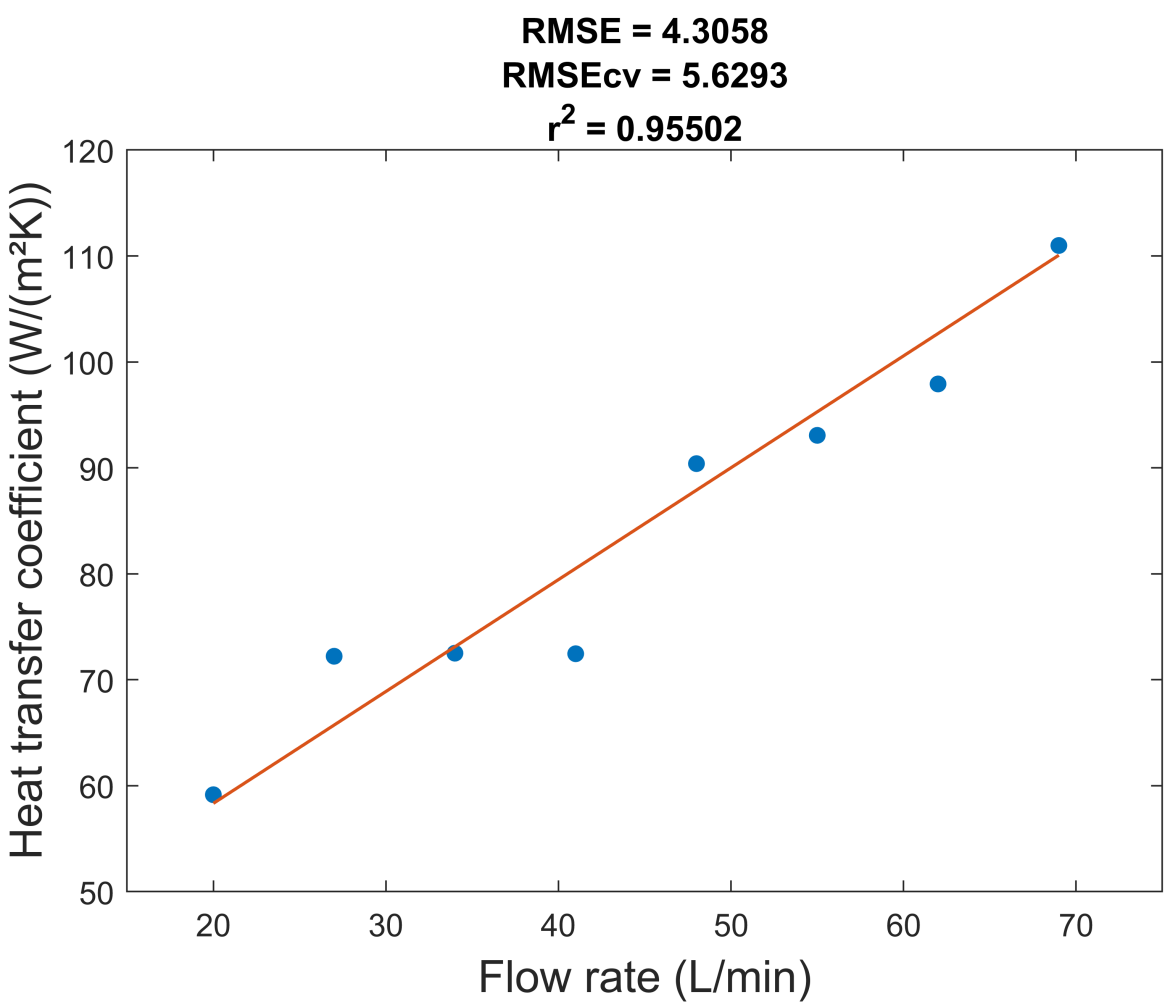
Linear regression model of volumetric gas flow rate in function of the heat transfer coefficient. The blue dots indicate experimental data, and the red solid line indicates the linear regression model. The linear regression had a slope of 71.11 E3 J/(m${}^{5}$K) and an intercept of 32.05 W/(m${}^{2}$K).

A linear regression model for determining the linear relationship between the gas flow rate and the heat transfer coefficient was constructed as described above, and regression coefficients were determined (Figure 5). $h_{a}$ was calculated to be 71.11 E3 J/(m${}^{5}$K), and $h_{b}$ was 32.05 W/(m${}^{2}$K). The RMSE was 4.3058 W/(m${}^{2}$K) and the RMSECV 5.6293 W/(m${}^{2}$K).

It must be noted that the product temperature was not directly predicted by the model developed in this work. The first reason is that model verification is significantly easier when considering the outer vial temperature, since this temperature can be directly verified using infrared camera measurements. Secondly, the product temperature is spatially different across the product layer, depending on the different phases of spin freezing. For example, during the crystal growth phase, the local temperature in the liquid layer will be the equilibrium temperature (i.e., 0 ${}^{{}^{\circ}}$C for liquid water). At the same time, the growing layer of solid ice is already cooling down below the equilibrium temperature as there is no longer any exothermic crystallization. Additionally, there is a temperature gradient across the product layer due to the cooling flux during the process. In essence, the product temperature cannot be defined or predicted as a single value, which is why the outer vial temperature was calculated in this model. It should be noted that the outer vial temperature is sufficient to obtain good process control, as it is representative for the average product temperature, and the product temperature can be calculated precisely at any location in the product layer using Equations (18) and (22).

**Figure 6:**
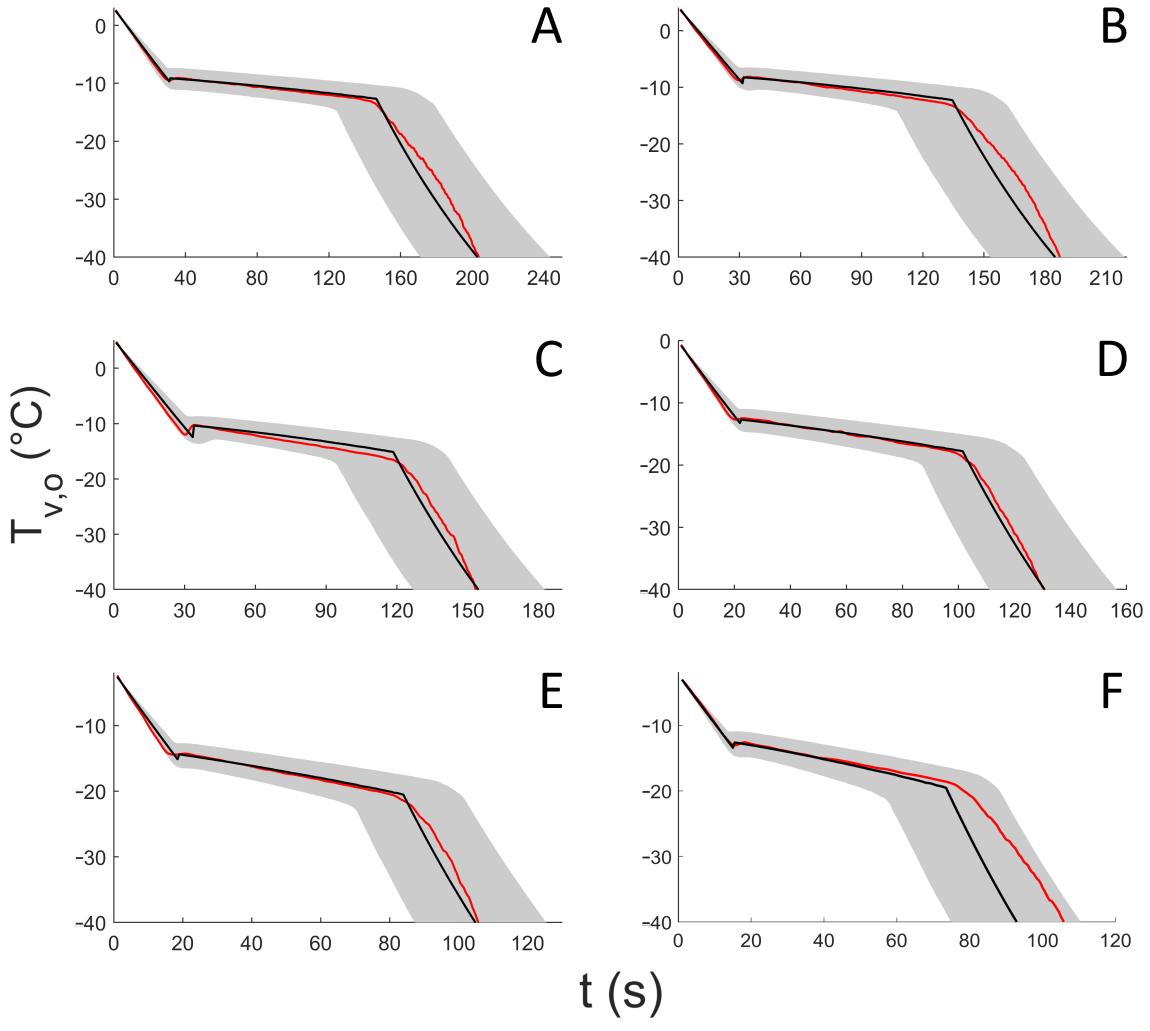
Uncertainty analysis of outer vial wall temperature profiles during cooling and freezing for 6 constant gas flow rates. The red solid lines represent outer vial wall temperature data from experiments as measured by the infrared camera. The black solid lines represent the model prediction of the outer vial wall temperature based on the model input parameters, assuming that no uncertainty is present on the model input parameters. The gray areas indicate the prediction interval of the model prediction of the outer vial wall temperature, taking the uncertainty in the model input parameters into account. Data of the following constant gas flow rates are displayed: A: 32 L/min, B: 44 L/min, C: 50 L/min, D: 56 L/min, E: 68 L/min, and F: 80 L/min.

The temperatures of the vial wall measured using the infrared camera and the calculated model prediction, including the model prediction uncertainty interval, are shown for six representative spin freezing runs in Figure 6. During the liquid cooling phase, $T_{v,o}$ decreased until nucleation occurs. It can be seen that the cooling rate during this first phase depends on the gas flow rate (Table 2). As the gas flow rate $\dot{V}$ increases, *h* also increases (Equation (13)). This results in a larger rate of heat transfer $\dot{Q}$ (Equation (9)) and subsequently, a higher cooling rate (Equation (28)).

Nucleation occurred at a measured (i.e., by the infrared camera) outer vial wall temperature between −15 °C and −9 °C. By taking the temperature gradient across the glass vial wall into account, this results in a product temperature at the inner vial wall at the point of nucleation between −2 °C and 0 °C (Table 2). The range of nucleation temperatures measured at the outer vial wall was larger than the actual range of nucleation temperatures. The reason for this is that the lower nucleation temperatures were generally the result of experiments at high gas flow rates. Here, the rate of heat transfer across the glass vial wall was high, resulting in a large temperature gradient (Equation (18)). This seemingly results in very low measured nucleation temperatures, while the temperature at the inside of the vial wall at the point of nucleation was still high and similar for all performed experiments.

It should be noted that nucleation is a stochastic event and was not controlled in this study. Interestingly, although uncontrolled, nucleation took place at relatively high temperatures (i.e., ≥−2 ${}^{{}^{\circ}}$C for all studied experimental conditions) compared to what is commonly seen in the literature, and the range wherein nucleation occurs appeared small (i.e., 2 ${}^{{}^{\circ}}$C) [4,18,31]. Taking into account that the rotating vials were not closed with stoppers, it is possible that a certain amount of “ice fog” was created, which caused early nucleation of the product. Controlled nucleation via ice fog is a known technique whereby cold nitrogen gas is introduced into the freezing environment, creating a fine mist of ice crystals from the moisture in the freezing chamber. These crystals then come into contact with the solution, inducing nucleation if the product temperature is sufficiently low [4,5,32]. In the case of spin freezing, since the gas used to cool the vials is very cold (i.e., −40 to −90 °C), it is possible that a mist of ice crystals is generated as mixing occurs with air containing moisture present in the freezing chamber, which then results in rapid nucleation when the product temperature drops below the equilibrium freezing temperature. Additionally, the mechanical agitation due to the spinning of the vial may be a reason for early nucleation. Indeed, Konstantinidis et al. described mechanical induction of nucleation, suggesting that vibrational disturbances may play a role in this case [33]. Low values and small ranges of supercooling as those seen here may be beneficial with regards to ice crystal size (i.e., large crystals are formed) and vial-to-vial variability, respectively [4]. However, this apparent controlled nucleation phenomenon during spin freezing and its effect on subsequent drying steps must be investigated in more detail. To this end, nucleation behaviour and potential controlled nucleation approaches during spin freezing are the topic of a future investigation.

Once nucleation had taken place, the product temperature increased until $T_{eq}$ for all studied conditions (Figure 6). The duration of the subsequent crystal growth phase depends on the rate of heat transfer. The experiments at higher gas flow rates had shorter crystal growth phases, as expected (Table 2). Again, as the gas flow rate $\dot{V}$ increases, $\dot{Q}$ also increases (Equation (9)), which subsequently results in faster crystallization of ice (Equations (20) and (21)). During the crystal growth phase, $T_{v,o}$ decreases slightly. As the crystal growth phase progresses, the ice layer forming on the inner vial wall grows. This results in an increase in the temperature gradient between the liquid product (i.e., at equilibrium temperature) and the inner vial wall (Equation (22)). Consequently, this explains the decreasing temperature over time at the outer vial wall (Equation (18)).

After completion of the crystal growth phase, indicated by a sharp temperature drop (i.e., measured as well as predicted by the model), the vial wall cooled down until the final temperature (i.e., −50 ${}^{{}^{\circ}}$C), at which point the process was considered complete. It should be noted that, in practice, there is a lower limit of this final temperature, determined by $T_{gas}$. In turn, $T_{gas}$ depends on the characteristics of the heat exchanger (e.g., cooling medium temperature) but also on the gas flow rate. As the gas flow rate decreases, $T_{gas}$ increases, since the slower-moving cold gas has more time to heat up when travelling from the heat exchanger to the gas nozzle (i.e., a distance of approximately 50 cm) and finally the vial. As the cooling process is driven by the difference between $T_{v,o}$ and $T_{gas}$, the vial wall can never cool below $T_{gas}$, as $\dot{Q}$ reaches a value of zero when $T_{v,o}$ equals $T_{gas}$. Similarly to the liquid cooling phase, the solid-phase cooling rate depends on the gas flow rate.

As seen in Figure 6, the model generally described the data well, as the experimental data obtained through infrared measurements consistently lay within the calculated prediction interval limits of the model predictions, for all tested gas flow rates.

### 3.2. Imposed Cooling Profile

**Figure 7:**
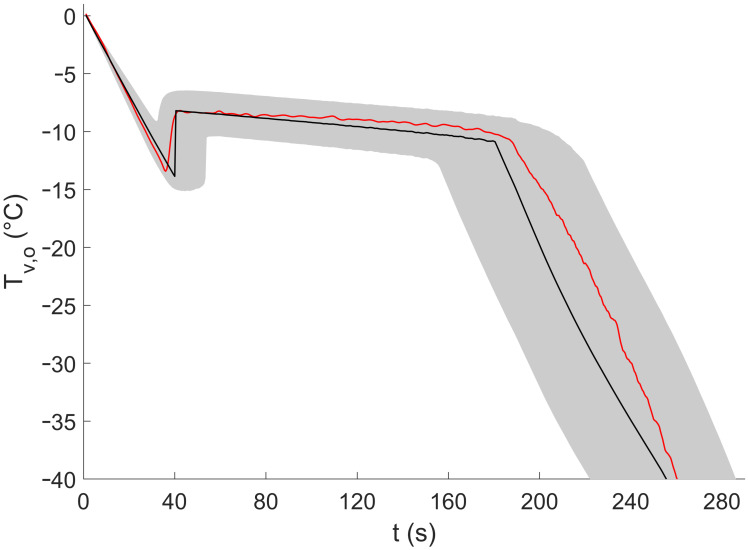
Uncertainty analysis of one representative outer vial wall temperature profile of the 20 imposed cooling profiles (i.e., which had liquid- and solid-phase cooling rates of 20 ${}^{{}^{\circ}}$C/min and a crystallization phase duration of 150 s). The red solid line represents the outer vial wall temperature data from the experiment as measured by the infrared camera. The black solid line represents the model prediction of the outer vial wall temperature based on the model input parameters, assuming that no uncertainty was present in the model input parameters. The gray area indicates the prediction interval of the model prediction of the outer vial wall temperature, taking the uncertainty in the model input parameters into account.

The uncertainty analysis of one representative-imposed cooling profile can be found in Figure 7. Similar to the experiments with constant gas flow rates, the experimental data (i.e., obtained using the infrared camera) for the imposed cooling experiments also fell within the uncertainty limits of the model prediction. The measured cooling rates during the liquid cooling and solid cooling phases were 22.19 ± 2.14 ${}^{{}^{\circ}}$C/min and 23.05 ± 2.64 ${}^{{}^{\circ}}$C/min, respectively (mean ± SD). The measured crystal growth duration was 151 ± 7.8 s (mean ± SD). Both cooling rates were slightly higher than the set value. As seen further (Section 3.3), the uncertainty in the heat transfer coefficient was very influential with regards to the overall model error. Hence, it is likely that the true values for the regression model parameters were higher than those calculated during the calibration. As a result, the calculated value of the gas flow rate (Equation (13)) was larger than the real required value necessary to obtain a cooling rate of 20 ${}^{{}^{\circ}}$C/min. Using a more elaborate calibration procedure (e.g., performing more calibration experiments resulting in more calibration points), it may be possible to reduce this systematic error. Although it does appear that the model slightly overestimated $T_{v,o}$ at higher gas flow rates during the liquid cooling phase, the data were still within the model prediction intervals. It should be noted that alternative control mechanisms such as closed control loops (e.g., proportional integral derivative control) can be used to control the cooling phases with significantly less uncertainty than the open-loop-model-based approach presented here. Using these control loops, measured temperature data would be fed into the control loop, which then outputs the required process settings (e.g., the gas temperature and the gas flow rate).

Contrary to batch freeze-drying, in spin freeze-drying it is possible to tightly control the rate of heat transfer during the freezing phase using the model developed in this work. Indeed, during batch freeze-drying, the rate of heat transfer during freezing is governed by the temperature difference between the vials containing the product and the temperature-controlled shelves. The temperature-controlled shelves are slow to react to changes in the set point temperature, while a change in the gas flow rate during spin freezing is near instantaneous. This allows for rapid changes in the rate of heat transfer at any point (e.g., immediately after nucleation) during the spin freezing process. As mentioned previously, since nucleation is stochastic, vials nucleate at different shelf temperatures during batch freeze-drying, resulting in different rates of heat transfer between vials during ice crystallization. In spin freezing, it is possible to compensate for the stochastic behaviour of nucleation by changing the rate of heat transfer after nucleation to a desired value, which allows for the manipulation of the freezing rate to obtain desired product characteristics such as dried layer resistance [3]. Spin freezing also has a broad range of possible freezing rates, limited only by the temperature and the flow rate of the used cooling gas. On the contrary, batch freezing is limited by the cooling speed of the temperature-controlled shelves (i.e., usually no faster than 1 ${}^{{}^{\circ}}$C/min). Many products that are sensitive to the freezing rate with regards to product integrity may benefit from this increased freezing rate range and level of control. In a recent work regarding the distribution of COVID-19 vaccines, the low freezing rate associated with batch freeze-drying was mentioned as a limitation that potentially causes phase separation, impairing product quality [34]. There have been similar findings in studies of other vaccines but also in studies of monoclonal antibodies and other protein-based products [35,36,37,38]. Additionally, the freezing rate is considered very important when freezing cell-based products such as microorganisms or mammalian-cell-based therapeutics, which are classes of products that are expected to become increasingly important in medicine [39,40,41,42,43].

### 3.3. Global Sensitivity Analysis

**Figure 8:**
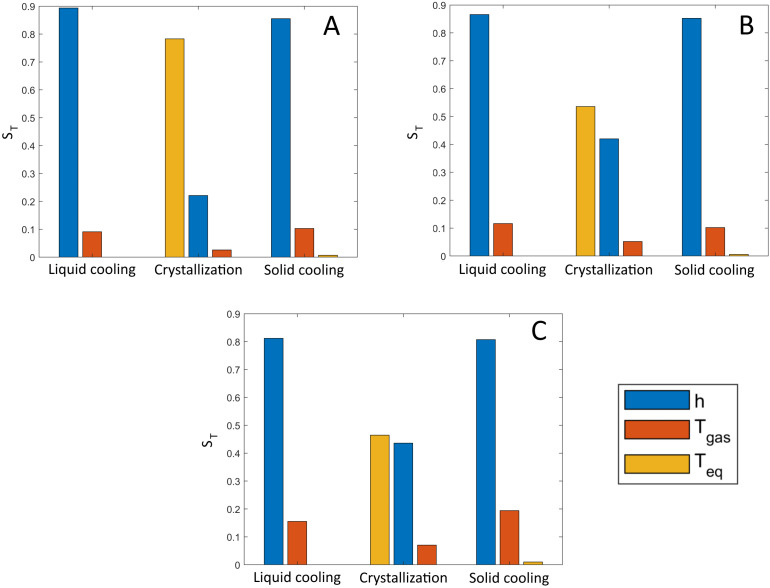
Global sensitivity analysis of the mechanistic model. Total order effect values $S_{t}$ are shown for every input parameter of the model during solid cooling, crystallization, and liquid cooling at different gas flow rates. Parameters with scores under 0.05 for every gas flow rate and phase of spin freezing are not shown. A: 20 L/min, B: 50 L/min, and C: 80 L/min.

In order to explain the results of the uncertainty analysis discussed above, a global sensitivity analysis was performed. In this way, the contribution of the uncertainty of the model input parameters to the overall model output uncertainty can be determined. As seen in Figure 8, the uncertainty in *h* was consistently the most influential on the model output uncertainty during the liquid cooling phase. After *h*, $T_{gas}$ follows as the second most important source of uncertainty of the model during the liquid cooling phase. *h* and $T_{gas}$ were responsible for 98.3%, 98.4%, and 98.0% of the prediction uncertainty for gas flow rates of 20, 50, and 80 L/min, respectively. The four remaining parameters (i.e., ${Ø}_{vial}$, $m_{vial}$, $m_{water}$, and $\dot{V}$) were considered as having a negligible impact on the uncertainty during the liquid cooling phase. In essence, the error on these four parameters was too small to have any noticeable impact on overall model uncertainty.

During the crystal growth phase, the uncertainty in the measurement of the equilibrium temperature had the most important impact for all gas flow rates. The equilibrium temperature is the starting point for $T_{v,o}$ during the crystal growth phase, resulting in a large impact on the uncertainty during this phase. However, $T_{gas}$ and *h* still have a relevant impact, especially at higher gas flow rates. Equation (25) shows how the thickness of the ice (i.e., the temperature gradient across this ice layer) and the rate of heat transfer at that time point must be taken into account when calculating $T_{v,o}$. Both of the aforementioned parameters influence the temperature gradient across the ice, explaining why *h* and $T_{gas}$ have an influence on uncertainty during the crystallization phase. However, the relative importance of the equilibrium temperature did seem lower with an increasing gas flow rate. A rising gas flow rate also resulted in an increase in the $\left(T_{v,o}-T_{gas}\right)$ term of Equation (9), as higher gas flow rates are associated with lower values of $T_{gas}$ (see above). $\left(T_{v,o}-T_{gas}\right)$ was subsequently multiplied by *h*, including its uncertainty. Therefore, higher gas flow rates result in a higher impact of the uncertainty in *h* on the overall model uncertainty relative to the contribution of $T_{eq}$. Similar to the liquid cooling phase, at least 96% of all variability was explained by the uncertainty in *h*, $T_{eq}$, and $T_{gas}$ for every gas flow rate.

Finally, during the solid cooling phase, the observations of the liquid cooling phase were still valid. Again, close to all model output uncertainty (i.e., >96%) was explained by the uncertainty in the regression model parameters, $T_{eq}$ and $T_{gas}$.

In essence, the GSA showed that the uncertainty in *h* was by far the most impactful on overall model uncertainty, followed by the uncertainty in $T_{gas}$, and, during the crystallization phase, the uncertainty in $T_{eq}$. The uncertainty in the remaining four parameters (i.e., ${Ø}_{vial}$, $m_{vial}$, $m_{water}$, and $\dot{V}$) had a negligible impact on the overall model uncertainty. Therefore, to reduce the model uncertainty, the focus should lie in the uncertainty of *h*. As mentioned above, using a more elaborate calibration procedure may help in this way to alleviate some of the model uncertainty.

## 4. Conclusions

Using in-line thermal imaging, it is possible to predict and control the product and vial temperature profiles during spin freezing using the mechanistic model developed in this work. The model was experimentally verified using an uncertainty analysis and was further characterized using a global sensitivity analysis. The uncertainty analysis was used to establish a prediction interval that contained the experimental data in every experimental verification run. The global sensitivity analysis was performed to determine the contribution of each model input parameter’s uncertainty to the uncertainty of the model output (i.e., the predicted temperature). The uncertainty in the heat transfer coefficient, the uncertainty in the gas temperature $T_{gas}$, and the uncertainty in the equilibrium temperature $T_{eq}$ were the most important parameters influencing the overall temperature prediction uncertainty. While the relative contribution of these three parameters differed depending on the phase of freezing and the gas flow rate, together they always explained at least 96% of the uncertainty in the model prediction. The remaining four parameters (i.e., ${Ø}_{vial}$, $m_{vial}$, $m_{water}$, and $\dot{V}$) were therefore considered to be of lesser importance regarding the model prediction uncertainty.

It was found that the calibration of the heat transfer coefficient has a large influence on the model uncertainty. Additionally, the applicability of the model was limited to the conditions where calibration was performed (e.g., the distance between the vial and the gas nozzle and the rotation speed of the vial). Therefore, to deal with these disadvantages, alternatives such as computational fluid dynamics or mechanistic calculation of the heat transfer coefficient should be investigated. To increase the applicability of the calibration approach, it will be investigated how different parameters of the spin freezing setup (e.g., the vial rotation speed) influence the regression model parameters. When the influence of the main parameters can be calculated, calibration data may be transferable to different spin freezing conditions, provided that the necessary adjustments are calculated.

In summary, a mechanistic model predicting the vial temperature during the spin freezing phase of a continuous freeze-drying process was developed and evaluated thoroughly. The results of the verification and subsequent analyses indicate a good process understanding. This model can be applied to control the spin freezing phase during continuous freeze-drying to overcome some of the disadvantages related to batch freeze-drying.

## Figures and Tables

**Table 1:** Input parameters and their uncertainty level for the UA and GSA.

Factor	Uncertainty Value	Reason of Inclusion
*h* (W/(m${}^{2}$K))	4.3058	RMSE on regression model
${Ø}_{vial}$ (m)	$1\times 10^{-4}$	Uncertainty of vial properties
$m_{vial}$ (kg)	$5\times 10^{-4}$	Uncertainty of vial properties
$m_{water}$ (kg)	$3\times 10^{-5}$	Measurement error
$\dot{V}$ (m${}^{3}$/s)	$6.67\times 10^{-6}$	Uncertainty of the gas flow rate measurement
$T_{eq}$ (${}^{{}^{\circ}}$C)	2	Uncertainty of the infrared temperature measurement
$T_{gas}$ (${}^{{}^{\circ}}$C)	2	Uncertainty of the gas temperature measurement

**Table 2:** Relevant experimental data from verification experiments.

Gas Flow Rate (L/min)	Cooling Rate Liquid Cooling Phase ${}^{{}^{\circ}}$C/min)	Cooling Rate Solid Cooling Phase ${}^{{}^{\circ}}$C/min)	Length Crystallization Phase (s)	$T_{\mathrm{nuc}}$ Outer Vial Wall ${}^{{}^{\circ}}$C)	$T_{\mathrm{nuc}}$ Inner Vial Wall ${}^{{}^{\circ}}$C)
20	18.5	14.3	161	−10.6	−1.8
26	24.6	25.7	134	−10.8	−0.4
32	25.0	28.6	123	−9.0	−0.6
38	27.4	26.3	115	−9.6	−0.3
44	25.8	26.7	116	−8.7	−0.9
50	33.6	54.5	87	−11.4	−2.0
56	38.7	67.1	80	−12.1	−0.5
62	53.8	79.3	65	−14.4	−0.9
68	47.0	91.3	66	−13.7	−0.5
80	46.5	82.9	60	−13.2	−0.9

## Data Availability

The data presented in this study are available upon request from the corresponding authors.

## Abbreviations

$T_{g}^{\prime }$	Glass transition temperature of the maximum freeze-concentrated drug solution (K)
$T_{e}$	Eutectic temperature (K)
ρ	Density of the solution (kg/m^3^)
$c_{p}$	Specific heat capacity of the solution (J/(kg K)) (Nakagawa et al.)
*k*	Thermal conductivity (W/(m K)) (Nakagawa et al.)
*T*	Temperature distribution of the system (K) (Nakagawa et al.)
$Q_{n}$	Total latent heat released due to ice nucleation (W) (Nakagawa et al.)
$Q_{c}$	Total latent heat released due to ice crystallisation (W) (Nakagawa et al.)
$\Delta H_{f}$	Latent heat of crystallization (J/kg) (Nakagawa et al.)
$k_{i}$	Nucleation rate (kg/(m^3^ s K)) (Nakagawa et al.)
$T_{s}$	Temperature in the supercooled liquid (K) (Nakagawa et al.)
ν	Freezing front velocity (m/s) (Nakagawa et al.)
*s*	Thickness of the undercooled zone (m) (Nakagawa et al.)
T′	Homogeneous undercooled temperature (K) (Nakagawa et al.)
$\chi_{ice}$	Fraction of ice suspended in the undercooled solution (Nakagawa et al.)
L*	Mean ice crystal size (m)
*a*	an empirical constant based on experimental data (ms/K) in Equation (6)
*R*	Freezing front rate (m/s)
*G*	Temperature gradient in the frozen zone (K/m)
$T_{e,lag}$	Thermocouple lag error (K)
$N_{t}$	Time constant (s)
$N_{t,a}$	Time constant regression model coefficient (1/s)
$N_{t,b}$	Time constant regression model coefficient (m${}^{-\frac{1}{2}}$s${}^{-\frac{1}{2}}$)
*w*	Velocity of the air flow (m/s)
$Q_{cryst}$	Total heat released due to crystallization of ice (J)
$\bar{Q}$	Mean rate of heat transfer (W)
$m_{water}$	Mass of water in the vial (kg)
*h*	Heat transfer coefficient (W/(m${}^{2}$K))
${Ø}_{vial}$	Diameter of the glass vial (m)
$h_{vial}$	Height of the glass vial (m)
$T_{v,o}$	Temperature at the outside of the glass vial wall (K)
$T_{gas}$	Temperature of the gas (K)

$\dot{Q}$	Rate of heat transfer (W)
$t_{cryst}$	Duration of the crystal growth phase (s)
$\dot{V}$	Volumetric gas flow (m${}^{3}$/s)
$h_{a}$	Regression coeffient (J/(m${}^{5}$K))
$h_{b}$	Regression error term (W/(m${}^{2}$K))
$C_{tot,w}$	Total heat capacity of the system before freezing (J/K)
dt	Length of the modelling time step (s)
$c_{p\;water}$	Specific heat capacity of water (J/(kg K))
$c_{p\;vial}$	Specific heat capacity of the vial glass (J/(kg K))
$m_{vial}$	Mass of the glass vial (kg)
$r_{sc}$	Radius of the nucleation ice crystal formation (m)
$r_{v,o}$	The outer vial radius (m)
$k_{glass}$	Thermal conductivity of the vial glass (W/(m K))
$T_{v,i}$	Temperature at the inside of the glass vial wall (K)
$th_{ice}$	Thickness of the ice layer (m)
$r_{v,i}$	Inner vial radius (m)
$k_{ice}$	Thermal conductivity of ice (W/(m K))
$T_{eq}$	Equilibrium freezing temperature (K)
$\chi_{nuc}$	Fraction of ice crystallized after nucleation
$C_{tot\;nucl}$	Total heat capacity of the volume below the equilibrium freezing temperature (J/K)
$T_{nucl}$	Nucleation temperature (K)
$V_{nucl}$	Volume below the equilibrium freezing temperature (m${}^{3}$)
$\rho_{water}$	Density of water (kg/m${}^{3}$)
$\rho_{ice}$	Density of ice (kg/m${}^{3}$)
$C_{tot,i}$	Total heat capacity of the system after freezing (J/K)
$c_{p\;ice}$	Specific heat capacity of ice (J/(kg K))
$m_{ice}$	Mass of ice (kg)
GSA	Global sensitivity analysis
UA	Uncertainty analysis
$S_{Ti}$	Total order effect
$C_{r}$	Cooling rate (K/s)
